# Higher Education

**Published:** 1890-04

**Authors:** 


### Editorial.

HIGHER education is the theme now agitating the public mind. You hear it from the pulpit, on the hustings and around the fireside. There is a charm in the word "higher." Nevertheless, the Legislature of the Empire State (New York) has a bill under consideration entitled "An act to repeal the statute requiring the preliminary education of medical students." There is a great probability that the bill will pass, and thus defeat, for a time, the grand purpose of science. This is an injurious measure, showing that politicians are ever ready to sacrifice anything to their greed for office. We earnestly hope that the Governor will show his usual pluck, and promptly veto the *repealing act*, should it pass. If *higher education* is needed to advance the interest of the common country, it cannot certainly be any more needed than the preparing students to enter upon the study of the medical sciences. But few States have passed laws requiring preliminary preparation to enable candidates to begin the study of medicine. Alabama is the pioneer in this interest among the Southern States. There is in Georgia a college law, not statutory, which really only recommends, for the public good, that young men qualify themselves to enter upon the study of medicine. We believe in *higher* education, but it should be *high* first, and then *higher*. We mean there should be a solid foundation in the elementary branches. We believe in thorough training at the very beginning.

